# Supplementary material for: CD11c+ myeloid cells are the predominant CD4+CCR5+ immune population in the foreskin and are increased in men with HIV-associated penile anaerobes
Source: bioRxiv. 2026 May 14:2026.05.11.724468. Preprint. [Version 1] doi: 10.64898/2026.05.11.724468 (PMC13192802; doi:10.64898/2026.05.11.724468)
Supplement: 1 [file NIHPP2026.05.11.724468V1-supplement-1.pdf]

bioRxiv preprint doi: https://doi.org/10.64898/2026.05.11.724468; this version posted May 14, 2026. The copyright holder for this preprint (which was not certified by peer review) is the author/funder, who has granted bioRxiv a license to display the preprint in perpetuity. It is made available under a CC-BY 4.0 International license.

Supplemental Tables & Figures

Supplemental Table 1. Antibodies used for immunofluorescence and histology

Target	1°/ 2°	Clone	Supplier	Host Species	Dilution	Fluorophore
CD3	1°	SP7	Abcam	Rabbit	Neat	None
CD4	1°	Polyclonal	R&D	Goat	1:10	None
CCR5	1°	*	*	Mouse	1:10	None
CD207	1°	Polyclonal	R&D	Goat	1:20	None
CD11c	1°	EP1347Y	Abcam	Rabbit	1:250	None
CD68	1°	C68/684	Abcam	Mouse	1:500	None
Mouse IgG[†]	2°	Polyclonal	Fisher	Donkey	1:400	Alexa Fluor
Rabbit IgG[†]	2°	Polyclonal	Fisher	Donkey	1:400	Alexa Fluor
Goat IgG[†]	2°	Polyclonal	Fisher	Donkey	1:400	Alexa Fluor

[†] Binds both heavy and light IgG chains (H + L)

* Monoclonal hybridoma generously provided by Dr. Matthias Mack (University of Regensburg, Germany)

Supplemental Table 2. Excitation & Emission Filters for Immunofluorescence Microscopy

Target	Leica Filter	Absorbance λ	Excitation Filter[a]	Emission λ	Emission Filter[a]
DAPI	CFP	358	436/20	461	480/40
Alexa Fluor 488	GFP	494	425/60	517	480 LP
Alexa Fluor 546	DSR	556	545/30	573	620/60
Alexa Fluor 647	Y5	650	620/60	665	700/75

LP long pass filter
λ wavelength, in nanometers
[a]Peak wavelength of light that passes through the filter and filter bandwidth, in nanometers

bioRxiv preprint doi: https://doi.org/10.64898/2026.05.11.724468; this version posted May 14, 2026. The copyright holder for this preprint (which was not certified by peer review) is the author/funder, who has granted bioRxiv a license to display the preprint in perpetuity. It is made available under a CC-BY 4.0 International license.

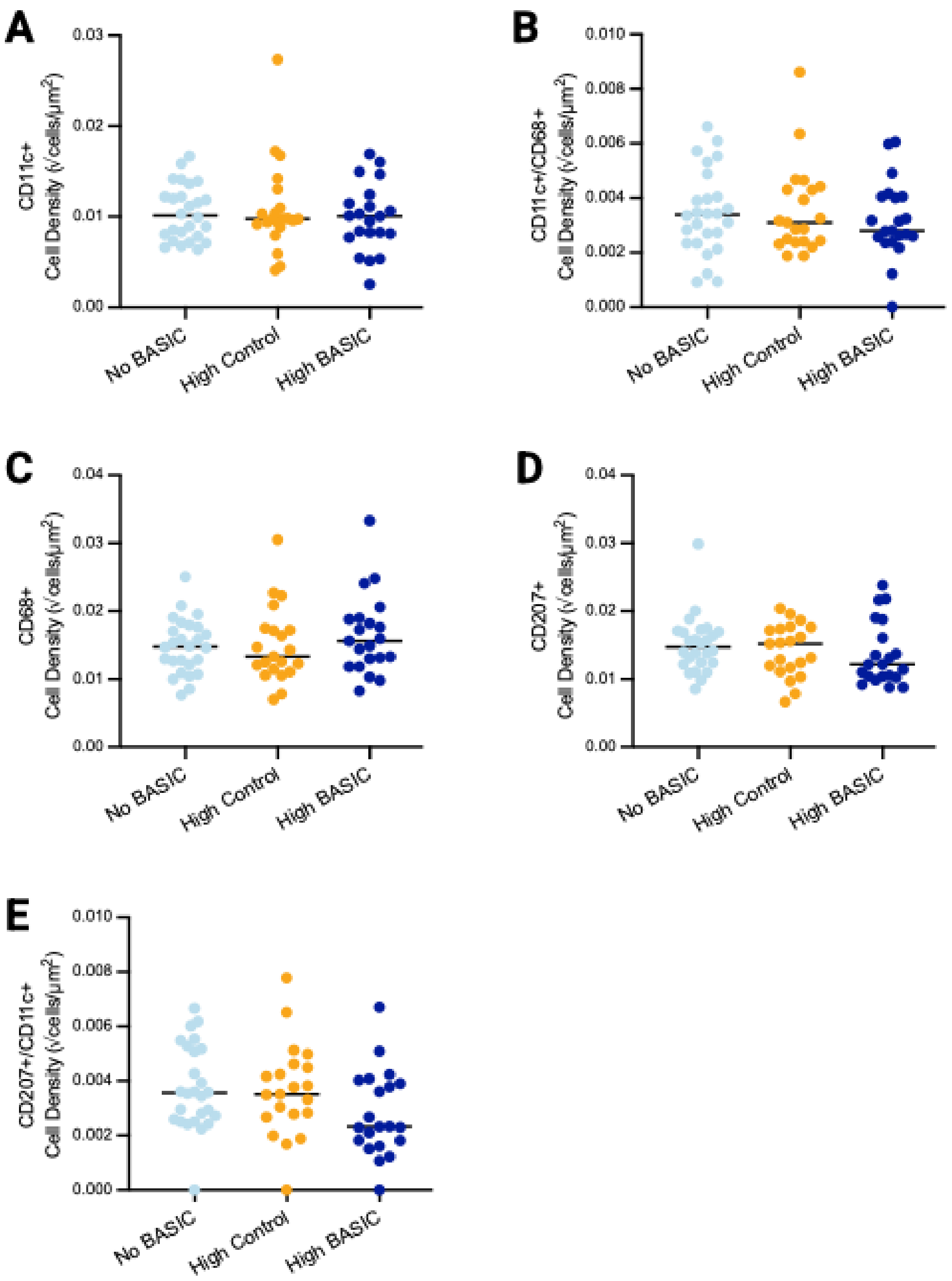

Supplemental Figure 1. Immune cell densities in the inner foreskin epidermis. CD11c+ (**A**), CD68+ (**B**), CD207+ (**C**), CD11c+CD68+ (**D**) co-expressing, and CD207+CD11c+ (**E**) co-expressing cells compared in the epidermis across High BASIC (n=21), High Control (n=21), and No BASIC (n=26) groups. Horizontal plot lines represent the median cell density (cells/μm²) for each group. Statistical significance was assessed using the Kruskal-Wallis test followed by Dunn's post-hoc test with Bonferroni correction, $\alpha = 0.05$. No significant differences were observed.
